# Next Generation Sequencing of Single Nucleotide Polymorphic DNA-Markers in Selecting for Intramuscular Fat, Fat Melting Point, Omega-3 Long-Chain Polyunsaturated Fatty Acids and Meat Eating Quality in Tattykeel Australian White MARGRA Lamb

**DOI:** 10.3390/foods10102288

**Published:** 2021-09-27

**Authors:** Shedrach Benjamin Pewan, John Roger Otto, Roger Huerlimann, Alyssa Maree Budd, Felista Waithira Mwangi, Richard Crawford Edmunds, Benjamin William Behrens Holman, Michelle Lauren Elizabeth Henry, Robert Tumwesigye Kinobe, Oyelola Abdulwasiu Adegboye, Aduli Enoch Othniel Malau-Aduli

**Affiliations:** 1 Animal Genetics and Nutrition, Veterinary Sciences Discipline, College of Public Health, Medical and Veterinary Sciences, Division of Tropical Health and Medicine, James Cook University, Townsville, QLD 4811, Australia; shedrach.pewan@my.jcu.edu.au (S.B.P.); john.otto@jcu.edu.au (J.R.O.); felista.mwangi@my.jcu.edu.au (F.W.M.); richard.edmunds@jcu.edu.au (R.C.E.); robert.kinobe@jcu.edu.au (R.T.K.); 2 National Veterinary Research Institute, Private Mail Bag 01 Vom, Plateau State, Nigeria; 3 Marine Climate Change Unit, Okinawa Institute of Science and Technology, 1919-1 Tancha, Onna-son, Okinawa 904-0495, Japan; roger.huerlimann@jcu.edu.au; 4 Centre for Sustainable Tropical Fisheries and Aquaculture and Centre for Tropical Bioinformatics and Molecular Biology, College of Science and Engineering, James Cook University, Townsville, QLD 4811, Australia; alyssa.budd@jcu.edu.au; 5 Centre for Red Meat and Sheep Development, NSW Department of Primary Industries, Cowra, NSW 2794, Australia; benjamin.holman@dpi.nsw.gov.au; 6 Gundagai Meat Processors, 2916 Gocup Road, South Gundagai, NSW 2722, Australia; MHenry@gmpgundagai.com.au; 7 Faculty of Veterinary and Agricultural Sciences, University of Melbourne, Melbourne, VIC 3010, Australia; 8 Public Health and Tropical Medicine Discipline, College of Public Health, Medical and Veterinary Sciences, Division of Tropical Health and Medicine, James Cook University, Townsville, QLD 4811, Australia; oyelola.adegboye@jcu.edu.au

**Keywords:** SNP, *FASN*, *SCD*, *FABP4*, IMF, FMP, eating quality, TAW MARGRA lamb, biopsy, n-3 LC-PUFA

## Abstract

Meat quality data can only be obtained after slaughter when selection decisions about the live animal are already too late. Carcass estimated breeding values present major precision problems due to low accuracy, and by the time an informed decision on the genetic merit for meat quality is made, the animal is already dead. We report for the first time, a targeted next-generation sequencing (NGS) of single nucleotide polymorphisms (SNP) of lipid metabolism genes in Tattykeel Australian White (TAW) sheep of the MARGRA lamb brand, utilizing an innovative and minimally invasive muscle biopsy sampling technique for directly quantifying the genetic worth of live lambs for health-beneficial omega-3 long-chain polyunsaturated fatty acids (n-3 LC-PUFA), intramuscular fat (IMF), and fat melting point (FMP). NGS of stearoyl-CoA desaturase (*SCD*), fatty acid binding protein-4 (*FABP4*), and fatty acid synthase (*FASN*) genes identified functional SNP with unique DNA marker signatures for TAW genetics. The *SCD g.23881050T>C* locus was significantly associated with IMF, C22:6n-3, and C22:5n-3; *FASN g.12323864A>G* locus with FMP, C18:3n-3, C18:1n-9, C18:0, C16:0, MUFA, and *FABP4 g.62829478A>T* locus with IMF. These add new knowledge, precision, and reliability in directly making early and informed decisions on live sheep selection and breeding for health-beneficial n-3 LC-PUFA, FMP, IMF and superior meat-eating quality at the farmgate level. The findings provide evidence that significant associations exist between SNP of lipid metabolism genes and n-3 LC-PUFA, IMF, and FMP, thus underpinning potential marker-assisted selection for meat-eating quality traits in TAW lambs.

## 1. Introduction

Eating quality is the single largest determinant of consumer acceptability and satisfaction with meat products. The eating and nutritional quality of lamb is influenced by intramuscular fat (IMF) content [1], fat melting point (FMP), tenderness, juiciness, flavor [2], and health-promoting omega-3 long-chain polyunsaturated fatty acids (n-3 LC-PUFA) that optimize retinal, maternal, and childhood brain functions while minimizing the risks associated with cardiovascular and chronic diseases [3,4].

In a recent review of the development, calibration, and validation of objective measurement technologies for carcass composition, lean, fat, and meat-eating quality traits in the Australian and New Zealand livestock industries, Gardner et al. [5] highlighted the inherent difficulties associated with the poor measurement of meat-eating quality and lean meat yield. Attempts to predict IMF [6,7,8], intramuscular connective tissue [9], composition and quality characteristics [10], tenderness, ultimate pH, and IMF content [11,12,13] from near infra-red based regression equations were characterized by low accuracy, inconsistency, and divergence between calibration and validation data. Such inaccuracies lead to lamb inefficiencies and an estimated annual value-chain wastage costs of $130 million to the Australian beef industry [5].

However, meat quality data can only be obtained after slaughter when selection decisions about the live animal are already too late. Carcass estimated breeding values [14,15], visual marbling score and meat imaging camera marbling systems [16], and dual X-ray absorptiometry scanner based computed tomography determined fat, lean muscle, and bone compositions of lamb carcasses [17] are all useful technological advancements, but still present precision problems due to low accuracy, and by the time an informed decision on the genetic merit for meat quality is made, the animal is already dead. In a study of associations of sire estimated breeding values and objective meat quality measurements with sensory scores in Australian lamb, Pannier et al. [18] confirmed the growing concerns that selecting for lean meat yield would reduce consumer eating quality and concluded that careful monitoring of selection programs is needed to maintain lamb eating quality. In an experimental trial to understand the impact of sire lean meat yield breeding value on carcass composition, meat quality, nutrient, and mineral content of Australian lamb, Knight et al. [14] concluded that to avoid deterioration in meat quality, the nutritional content of lamb and fresh meat color, Australian sheep producers will need to incorporate other aspects of meat quality when selecting sires with increased lean meat yield. To date, the use of conventional laboratory-based fat extraction, ‘slip point’ and gas chromatography methods still remain the most accurate techniques for not only measuring IMF, FMP, and n-3 LC-PUFA, but also for predicting consumer acceptance of beef and sheep meat [19]. Herein, we report for the first time, a combination of an innovative and minimally invasive *longissimus dorsi thoracis et lumborum* muscle biopsy sampling of Tattykeel Australian White (TAW) sheep exclusive to MARGRA lamb brand, laboratory-based IMF, FMP, and fatty acid analyses, and advanced genomics technique of next-generation sequencing (NGS) of single nucleotide polymorphisms (SNP) of lipid metabolism genes for directly quantifying the genetic worth of live lambs for health-beneficial n-3 LC-PUFA, IMF, and FMP. The primary objective was to conduct a NGS of stearoyl-CoA desaturase (*SCD*), fatty acid binding protein-4 (*FABP4*), and fatty acid synthase (*FASN*) lipogenic genes to identify functional SNP that provide unique DNA marker signatures for TAW genetics, breeding, and selection programs for meat-eating quality. The hypothesis tested was that *significant associations exist between SNP of lipid metabolism genes and n-3 LC-PUFA, FMP, and FMP underpinning potential marker-assisted selection for meat-eating quality traits in TAW lambs*.

## 2. Materials and Methods

### 2.1. Animals and Experimental Design

**Figure 1:**
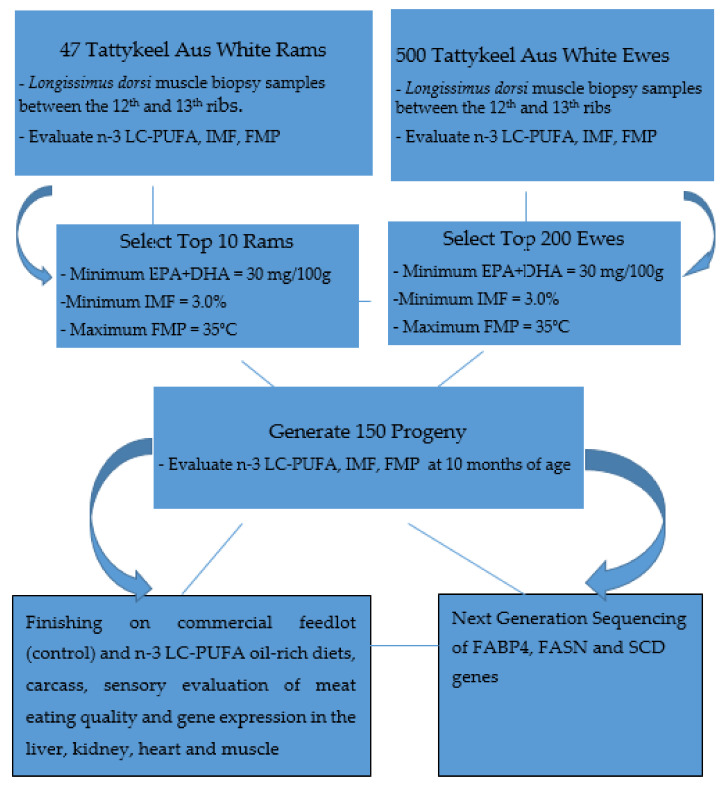
Experimental design for the selection, breeding, and evaluation of n-3 LC-PUFA, IMF, and FMP in Tattykeel Australian White sheep.

The experimental design for the selection, breeding, and evaluation of n-3 LC-PUFA, IMF, and FMP in Tattykeel Australian White (TAW) sheep is shown in Figure 1.

Three composite generations—parental, first (F_1_), and second (F_2_) composite generations of lambs were bred, raised, and maintained under the same management at the Tattykeel Australian White Stud in Black Springs, Oberon, New South Wales, Australia. The parental composite generation comprised 47 rams mated to 500 ewes after evaluating their *longissimus dorsi thoracis et lumborum* muscle biopsy samples for health-beneficial n-3 LC-PUFA, IMF, and FMP with minimum thresholds set at 30 mg/100 g, 3.0%, and 35 °C, respectively. The top 10 rams and 200 ewes were selected and mated to generate 150 progeny whose muscle biopsy samples were laboratory tested for n-3 LC-PUFA, IMF, FMP, and genomic DNA sequenced at 10 months of age prior to being finished at a commercial feedlot. The Poll Dorset and Texel were used as positive control and the Rambouillet as the negative control in assessing extracted genomic DNA, polymerase chain reaction products, and next-generation sequencing procedures in the laboratory. Details of the muscle biopsy procedure and laboratory analyses of IMF, FMP, and fatty acid composition had already been published [2] and are only briefly summarized below.

### 2.2. Muscle Biopsy Sampling Procedure

The biopsy procedure for sampling the *Longissimus dorsi* muscle from the 12th–13th ribs was first described in cattle [20] and modified in sheep [2]. Pewan et al. [2] published the details of the biopsy procedure in sheep, and these will not be repeated here.

### 2.3. Determination of Intramuscular Fat

Details of the procedures for laboratory analysis of intramuscular fat were published by Pewan et al. [2], Holman et al. [21], and Flakemore et al. [22] and will not be repeated here.

### 2.4. Determination of Fat Melting Point

Details of the laboratory analysis of fat melting point were published by Pewan et al. [2], Holman et al. [21], and Flakemore et al. [22] and needless to repeat herein.

### 2.5. Determination of Fatty Acid Composition

Fatty acid composition including n-3 LC-PUFA analysis of *Longissimus dorsi* muscle biopsy samples was analyzed by means of gas chromatography–mass spectrophotometry procedure described in detail by Malau-Aduli et al. [23] based on modified Bligh and Dyer [24], Miller et al. [25], and Clayton [26] methods. Details were published by Pewan et al. [2].

### 2.6. Blood Collection and Genomic DNA Extraction

About 10 mL of blood was collected from Tattykeel Australian White, Poll Dorset, and Texel (positive control) lambs of the same age and under the same management conditions by jugular venipuncture into vacutainers containing EDTA. Blood samples were stored at −80 °C until ready for genomic DNA (gDNA) extraction. gDNA was extracted from 2 mL of blood using NucleoSpin Blood Kits (Macherey-Nagel GmbH and Co. KG, Neumann-Neander-Str. 6-8. 52355 Duren, Germany) according to the manufacturer’s protocol. gDNA yield was quantified with a NanoDrop ND-1000 spectrophotometer (NanoDrop, Thermo Fisher Scientific Australia Pty Ltd., Scoresby, Victoria, Australia).

### 2.7. Primer Design

#### 2.7.1. *FASN*, *FABP4*, and *SCD* Primers

All primers were designed using Geneious Prime Software Program 2020 v.2.2 (http://www.geneious.com). A targeted candidate gene approach of lipid metabolism genes (*FASN*, *FABP4,* and *SCD*) was utilized. Single coding sequences of each gene deposited in the National Center for Biotechnology Information (NCBI) database (Genbank) of *FASN*, *FABP4,* and *SCD* of *Ovis aries* breed were used as reference points. In order to amplify the 18 kb of the *FASN* gene (Accession Number: NC_040262.1), a long-range PCR approach was used to split the gene sequence into 3 overlapping fragments of 8.5 kb each (*FASN*1, *FASN*2, and *FASN*3), comprising approximately 91% of the total gene sequence. For the 4 kb *FABP4* (NC_040260.1) and 12 kb *SCD* (NC_040273.1) gene fragments, a single primer set was designed as shown in Table 1. All primers were synthesized at Integrated DNA Technologies Pte. Ltd., Melbourne, Australia (Itddna.Com (accessed on 12 June 2021)).

#### 2.7.2. Long-Range PCR

Due to the different fragment lengths and DNA composition, it was necessary to use 3 different long-range PCR approaches to amplify the *FASN*, *FABP4,* and *SCD* genes. During optimization, all 3 approaches were tested for all 3 genes, but only the best performing combinations were utilized.

#### 2.7.3. *FASN* Gene

**Figure 2:**
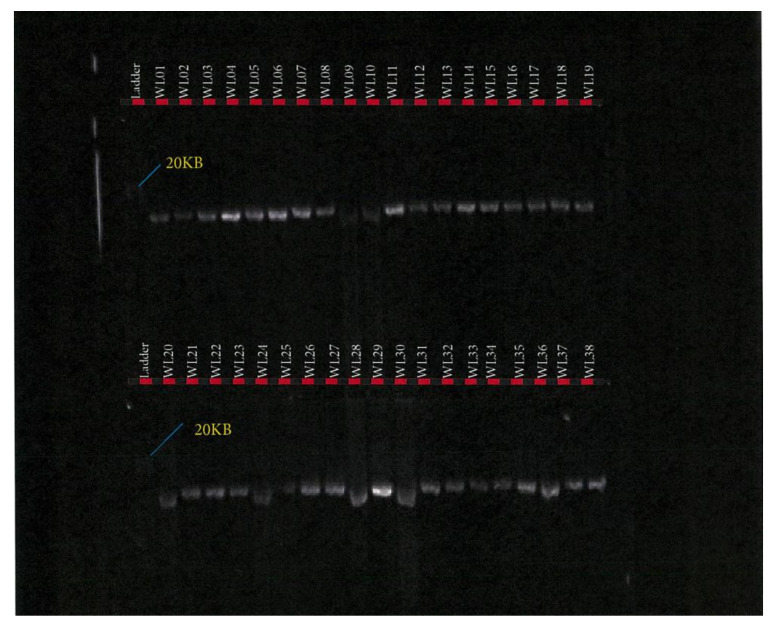
*FASN* fragment 1 PCR product in Tattykeel Australian White (WL), Poll Dorset (PD), and Texel (TX) lambs.

**Figure 3:**
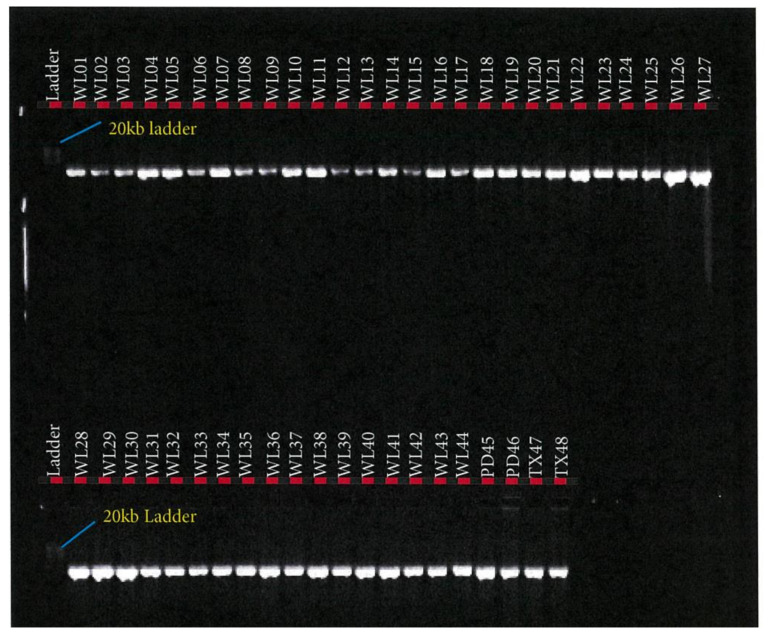
*FASN* fragment 2 PCR product in Tattykeel Australian White (WL), Poll Dorset (PD), and Texel (TX) lambs.

**Figure 4:**
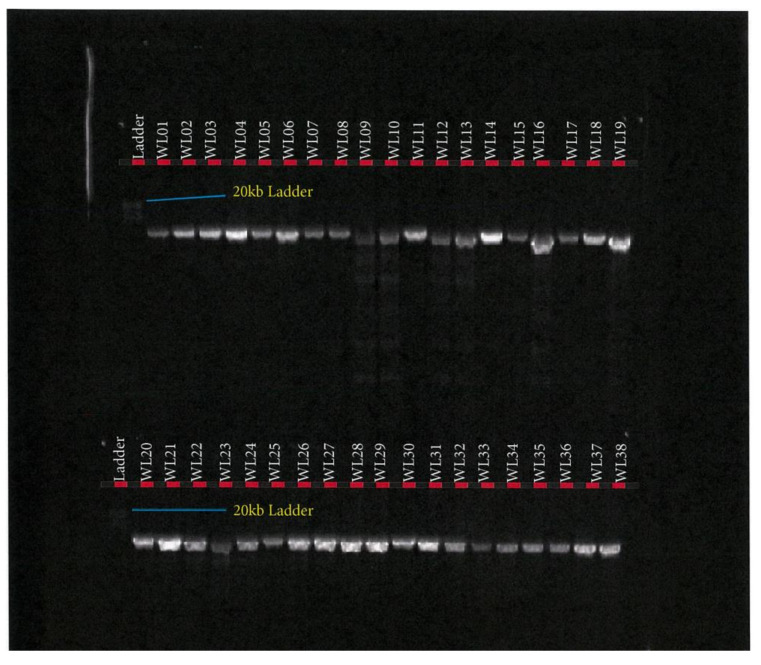
*FASN* fragment 3 PCR product in Tattykeel Australian White (WL), Poll Dorset (PD), and Texel (TX) lambs.

*FASN* PCR amplification assay was performed using the TakaRa PrimeSTAR GXL Master Mix (TaKaRa Bio Inc., Kusatsu, Shiga, Japan). PCR reaction assay was set up in a total volume of 50 µL containing 10 µL of 5× TakaRa PrimeSTAR GXL Buffer, 200 μM of TaKaRa dNTP Mixture, 1.25 units of TaKaRa PrimeSTAR GXL DNA Polymerase, 0.2 μM of each primer (IDT, Melbourne, Australia), and 100 ng of DNA template. PCR was performed in a SimpliAmp™ Thermal Cycler (Thermofisher Scientific, Melbourne, Australia), in a 2-step protocol using the following conditions: 98 °C initial denaturation for 1 min (1 cycle); 98 °C denaturation for 10 s; 68 °C annealing/extension for 10 min for 30 cycles. PCR success was checked in 0.8% agarose gel electrophoresis as depicted in Figure 2, Figure 3 and Figure 4.

#### 2.7.4. *FABP4* and *SCD*

**Figure 5:**
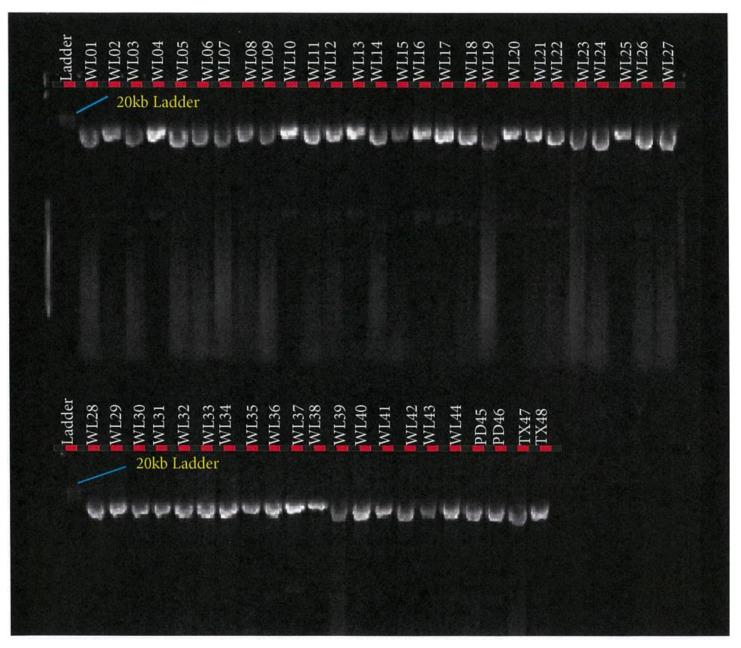
*SCD* PCR product in Tattykeel Australian White (WL), Poll Dorset (PD), and Texel (TX) lambs.

**Figure 6:**
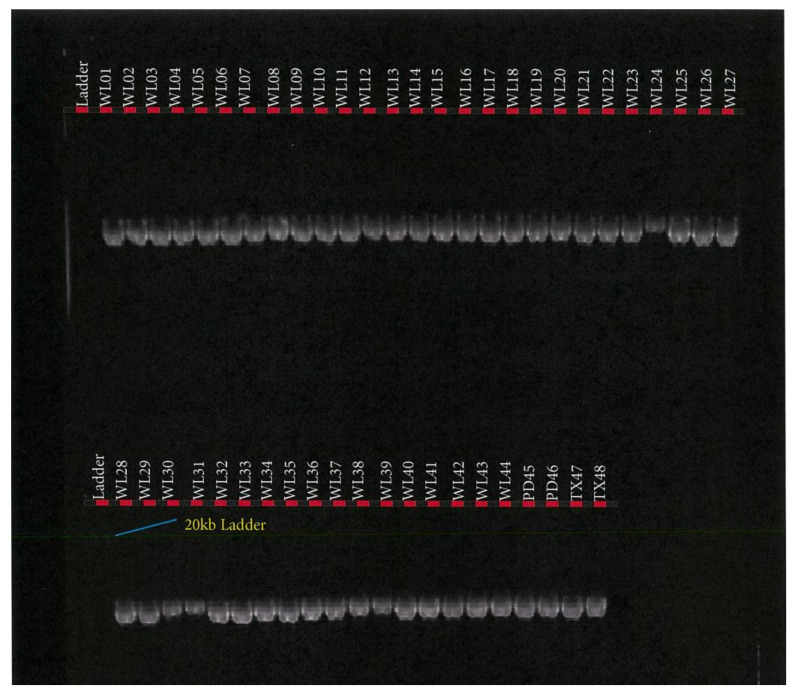
*FABP4* PCR product of Tattykeel Australian White (WL), Poll Dorset (PD), and Texel (TX) lambs.

For the FAPB4 gene, Platinum™ SuperFi™ II PCR Master Mix (Thermofisher Scientific, Australia) was used, while for the *SCD* gene, Hot Start II High-Fidelity PCR Master Mix (Thermofisher Scientific, Australia) was used under the same PCR conditions. The amplification reactions were performed in a total volume of 50 µL containing 25 µL of 2× Platinum™ SuperFi™ II PCR Master Mix or Phusion Hot Start II High-Fidelity PCR Master Mix (Thermofisher Scientific, Australia), 0.5 µM of each primer (IDT, Australia), and 100 ng of DNA template. PCR was performed in a SimpliAmp™ Thermal Cycler (Thermofisher Scientific, Australia), in a 3-step protocol, using the following conditions: 98 °C initial denaturation in 1 min (1 cycle); 98 °C for denaturation 15 s; 60 °C (*FABP4*)/and 65 °C (*SCD*) annealing for 15 s; 72 °C extension for 9 min; 72 °C final extension for 9 min; 4 °C hold for 35 cycles. PCR success was checked in 0.8% agarose gel electrophoresis, as depicted in Figure 5 and Figure 6.

### 2.8. PCR Clean-Up

Sera-Mag™ SpeedBeads was prepared according to Faircloth et al. [27] and used to clean the PCR products using a Zephyr NGS Workstation (Caliper Lifesciences, Perkin-Elmer) and quantified using a Promega dsDNA Quantifluor System Kit (Ref: E2670, 00002484139) on an Enspire Workstation (Perkin-Elmer). The 5 different PCR products were pooled at approximately 0.4 nM to ensure even coverage during sequencing using Quantifluor dsDNA System (Promega, Madison, WI, USA). The products were normalized to 2 ng/µL using 10 mM Tris-HCl (pH 8.0). Final dilution to 0.2 ng/µL with 10 mM Tris-HCl (pH 8.0) was conducted in preparation for library preparation and final accuracy checks using the Illumina Nextera^XT^ DNA.

### 2.9. Library Preparation, Quantification, Normalization, and Sequencing

Libraries were prepared using Nextera XT DNA Library Prep kit (Illumina, CA, USA) in accordance with the manufacturer’s protocols using the recommended input of 5 µL of 0.2 ng/µL gDNA per sample. This was followed by Sera-Mag™ SpeedBeads purification using 0.6× beads and 2 washes using 80% ethanol to select fragments > 250 bp and remove unincorporated adapters. Each DNA library fragment size and concentration was determined using Agilent High Sensitivity D5000 reagents and ScreenTape on the Tape Station 4200 Instrument (Agilent Technologies, Santa Clara, CA, USA) according to the Agilent assay quick guide. Additionally, all individual libraries were quantified using QuantiFluor^®^ dsDNA System (Promega, Madison, WI, USA) to give an additional concentration estimate. The resultant size and concentration data from Tape Station and Quantifluor system were used to normalize each library to 4 nM by diluting with 10 mM Tris-HCl (pH 8.5) prior to pooling. An equal volume of 5 µL was pooled and sequenced on an Illumina MiSeq benchtop sequencer, using a 500-cycle MiSeq Reagent Nano Kit v2 with a 10 pM input and 10% PhiX spike-in.

### 2.10. Bioinformatics and Next Generation Sequencing Data Analysis

Genomic data analysis was performed using commercial bioinformatics program Geneious Prime software program 2020 v.2.24 (http://www.geneious.com (accessed on 12 June 2021)) to analyze the sequences. The following reference sequences deposited in the NCBI database were used for comparative analysis: NC_040262.1, NC_040260.1, and NC_040273.1 for *FASN*, *FABP4,* and *SCD* genes, respectively. Next Generation Sequenced data were retrieved from Illumina Dashboard-BaseSpace Sequence Hub (https://basespace.illumina.com/dashboard (accessed on 15 July 2021)) as paired read data in 2 separate forward and reverse read lists in FASTQ format. The retrieved raw reads were subjected to quality control measures. Reads were trimmed and adapters removed using the BBDuk trimmer in Geneious Prime 2020 v.2.2 with the default setting for paired-end reads. The Quality (Q) value of Phred score was set at 20 to improve sequenced data and increase the likelihood of calling true SNPs to 99%. Short reads with a minimum length of 20 bp were discarded, resulting in clean reads. Regions of low coverage were excluded when calling SNPs using the Annotate and Predict → Find Low/High Coverage. The reads were mapped to reference in Geneious. The reference sequences were retrieved from NCBI database (Genbank) of *FASN*, *FABP4,* and *SCD* of *Ovis aries* breed. The Sensitivity was set on the Medium Sensitivity/Fast and Fine-Tuning (iterate up to 5 times) option selected to improve the results by aligning reads to each other in addition to the reference sequence. Major allele frequencies from the next-generation sequence data based on observed and expected genotypes were computed using the Hardy–Weinberg equilibrium principle as described by Graffelman et al. [28].

### 2.11. Statistical Analyses

All statistical analyses of the associations between detected SNP of the 3 genes and meat-eating quality traits were performed using R statistical software version 3.6.3 [29]. Linkage disequilibrium as an index of non-random association between alleles of different loci, was estimated as the difference between the frequency of gametes carrying the pair of alleles A and B at two loci (pAB) and the product of the frequencies of those alleles (pA and pB), D_AB_ = pAB − pApB, where the allele pair AB is a haplotype and pAB is the haplotype frequency [30]. Major and minor allele frequencies were computed, and the Hardy–Weinberg Equilibrium was tested using the chi-square test. Pearson’s residual correlation analysis was carried out to examine the relationships between genomic variants and meat quality traits (FA, FMP, and IMF). Linear mixed models procedure was used to investigate differences in FMP, IMF, and fatty acid profiles of the TAW lambs due to *FABP4*, *SCD,* and *FASN* variants fitting the fixed effect of allele substitution for individual SNP and random effect of animal (for pedigree) accounting for composite generation effects. Functional allele mutations at the coding regions of identified *FABP4*, *SCD,* and *FASN* loci were statistically analyzed for association with FMP, IMF, and fatty acids. Least-square means were compared using the Tukey-adjusted multiple comparisons test. The full statistical model was:

$$Y_{\mathrm{ij}}=µ+\alpha_{i}+\gamma_{1}{\mathrm{FA}}_{\mathrm{ij}}+\gamma_{2}{\mathrm{SC}}_{\mathrm{ij}}+\gamma_{3}{\mathrm{SK}}_{\mathrm{ij}}+e_{\mathrm{ij}}$$

where Y_ij_ = dependent variable (FMP, IMF, FA) of jth TAW of ith composite generation, µ = overall mean, α_i_ = effect of the ith composite generation, FA = the genotype *FASN* (AA, GA and GG), SC = the genotype *SCD* (CC, CT and TT), SK = the genotype *FABP4* (GG, GA and AA), γ = effect of the genotype, and e_ij_ = residual error.

## 3. Results

This study of *SCD*, *FASN,* and *FABP4* lipogenic genes SNP in TAW lamb muscle biopsy samples bred, selected, and evaluated as per the experimental design shown in Figure 1, was based on the Geneious-designed primers whose sequences are presented in Table 1 and successful polymerase chain reactions (PCR) products are presented in Figure 2, Figure 3, Figure 4, Figure 5 and Figure 6.

### 3.1. SCD, FASN, and FABP4 Gene SNP Variants and Genotypes

Using the Poll Dorset and Texel as positive controls, and Rambouillet as negative controls, eight *SCD* gene SNP loci (g.23880613A>G; g.23881050T>C; g.23883280G>A; g.23885910C>A; g.23887165A>G; g.23888763C>T; g.23889346T>G; g.23890209T>C) with major allele frequencies ranging from 0.53 to 0.93 were identified as depicted in Table 2. It was evident from Table 2 that TAW lambs were all heterozygous at three loci (g.23881050T>C, g.23883280G>A g.23885910C>A) in the parental, first, and second composite generations, thereby presenting a genetic divergence from the homozygous variants seen in the Poll Dorset, Texel and Rambouillet controls.

As depicted in Table 3, nine functional SNP covering 91% of the *FASN* gene sequence were identified. The genotypes at the nine loci were all the same in TAW, indicating a consistent heredity pattern from the composite TAW parents to the first and second generations, which were all distinguishable from the Rambouillet negative control breed. For the *FABP4* gene, three SNP loci were genotyped with major allele frequencies ranging from 0.50 to 0.97 (Table 4).

### 3.2. Correlations between SCD, FASN, and FABP4 Gene SNP, FMP, IMF, and Fatty Acids

**Figure 7:**
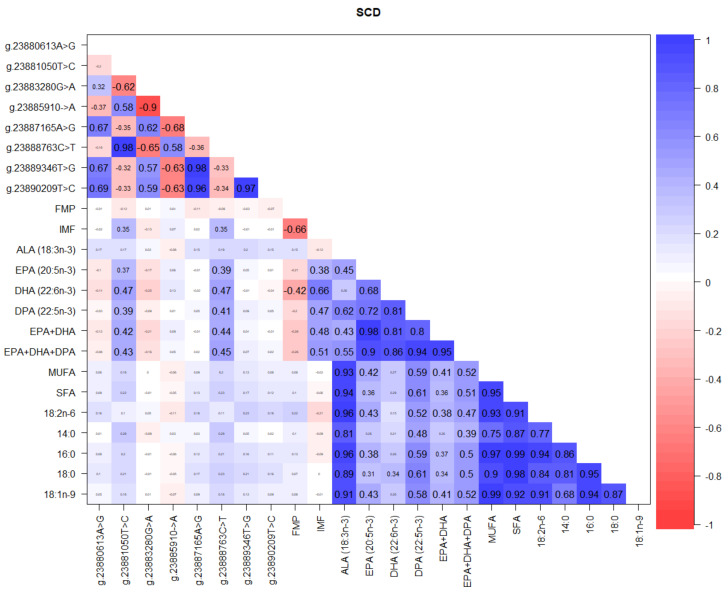
Correlations between *SCD* gene SNP loci, IMF, FMP, and fatty acids in TAW lambs.

Figure 7 shows significant correlations between detected *SCD* SNP loci, several fatty acids and other meat-eating quality traits. Among *SCD* SNP loci, the highest correlations of 0.98 were observed between g.23888763C>T and g.23881050T>C; g.23889346T>G and g.23887165A>G. Moderate correlations between health-promoting n-3 LC-PUFA (EPA, DHA, and DPA), and g.23888763C>T and g.23881050T>C loci ranging from 0.37 to 0.47 were observed. IMF was moderately to highly correlated with n-3 LC-PUFA (0.38–0.66), while FMP was negatively correlated with IMF (−0.66) and DHA (−0.42). Among the different fatty acids and their summations, very high correlations of up to 0.99 were evident (Figure 7).

**Figure 8:**
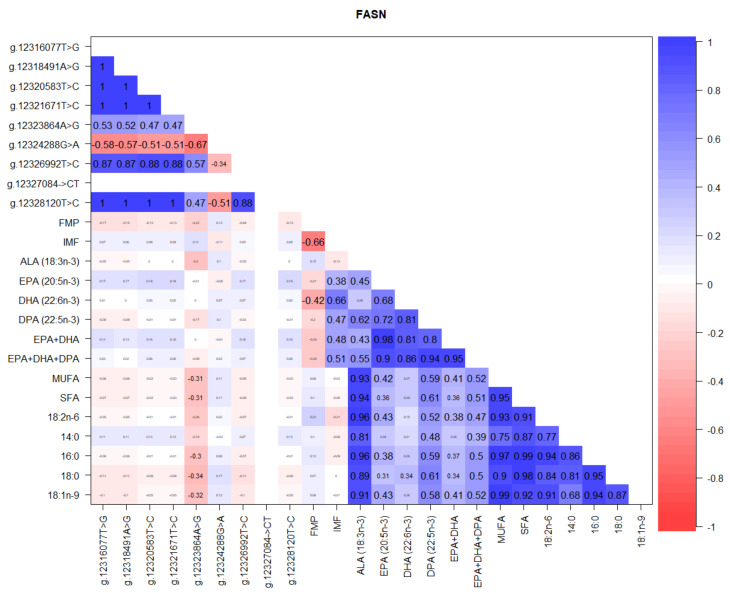
Correlations between *FASN* gene SNP loci, IMF, FMP, and fatty acids in TAW lambs.

Figure 8 shows that among *FASN* gene SNP, there were highly significant correlations between the loci, while correlations between the g.12323864A>G locus and most fatty acids were negative, ranging from −0.3 to −0.34. Negative correlations between IMF and FMP (−0.66) and DHA (−0.42) were also observed, while the highest positive correlations were between the various fatty acids (Figure 8).

**Figure 9:**
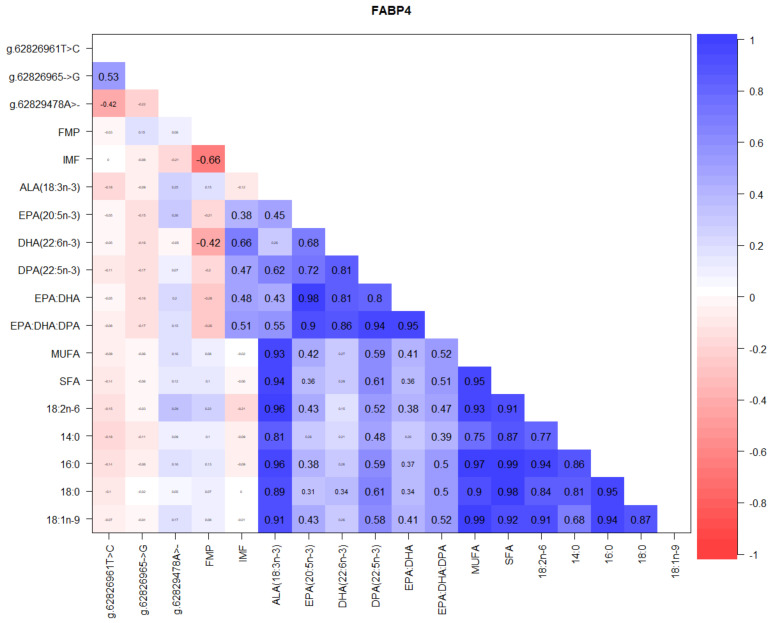
Correlations between *FABP4* gene SNP loci, IMF, FMP, and fatty acids in TAW lambs.

Figure 9 shows that among *FABP4* gene SNP, the highest correlation of 0.53 was between the loci g.62826965C>G and g.62826961T>C, while a negative correlation of −0.42 was observed between g.62826965C>G and g.62829478A>T. Consistently positive correlations between IMF and n-3 LC-PUFA of up to 0.66 with DHA, 0.47 with DPA, and 0.38 with EPA were also observed, while the highest positive correlations were among the various fatty acids and their summations (Figure 9).

### 3.3. Associations between SCD, FASN and FABP4 SNP, FMP, IMF, and Fatty Acids

Descriptive statistics of mean, standard deviation, and coefficient of variation of the meat quality traits and full suite of fatty acids breakdown are presented in Table 5. FMP had a mean of 33.65 °C with a standard deviation of 2.74 and coefficient of variation of 8.14%, while IMF averaged 4.43% with a standard deviation of 1.31 and coefficient of variation of 29.58%.

Table 5 also shows that the *SCD g.23881050T>C* SNP was significantly associated with IMF (*p* < 0.0089) and DHA (*p* < 0.0111), while *FABP4* g.62829478A>G SNP was associated with only IMF (*p* < 0.0539). The *FASN g.12323864A>G* SNP was associated with FMP (*p* < 0.0544), ALA (*p* < 0.0033), MUFA (*p* < 0.0025), SFA (*p* < 0.0025), C18:2n-6 (*p* < 0.0138), C16:0 (*p* < 0.0039), C18:0 (*p* < 0.0012) and C18:1n-9 (*p* < 0.0023) fatty acids (Table 5).

### 3.4. Tukey-Adjusted Multiple Comparison Tests for Significant SNP, FMP, IMF, and Fatty Acids

As depicted in Table 6, Tukey-adjusted multiple genotype comparison tests at the *SCD g.23881050T>C* SNP locus confirmed significant differences where the homozygous TT genotype had the highest DHA (11.00 ± 2.34 mg/100 g), IMF (5.43 ± 0.516%), and DPA (27.1 ± 3.26 mg/100 g) compared to the CC genotype with the lowest DHA (7.00 ± 2.11 mg/100 g), IMF (3.98 ± 0.312%), and DPA (17.9 ± 6.81 mg/100 g). The heterozygous genotype CT had intermediate DPA (7.64 ± 2.09 mg/100 g), IMF (4.39 ± 0.287%), and DPA (19.4 ± 6.74 mg/100 g) that were in-between the highest and lowest values (Table 6).

There were many more significant genotype variations at the *FASN g.12323864A>G* SNP mutation that was associated with FMP, ALA, MUFA, SFA, C18:2n-6, C18:1n-9, C18:0, and C16:0, in which the homozygous genotype GG had the highest values compared to the lowest values in AA genotype for all variables, with the exception of C18:2n-6 that was lowest in the heterozygous GA genotype (Table 6). In contrast, at the *FABP4* g.62829478A>G SNP locus, only IMF variation tended towards significance between the genotypes (*p* < 0.06).

## 4. Discussion

It is well-established that DNA-based inheritance enables the transmission of selected phenotypes across generations either without changes in the DNA sequence through epigenetic inheritance [31] or through functional mutations involving changes in only one base pair (single nucleotide polymorphisms—SNP). Through next-generation sequencing, SNP are valuable for detecting genetic variability and genomic prediction in sheep breeding programs [32], developing breed-specific DNA markers for breed identification [33,34], animal productivity [35], parentage assignment [36,37], forensics [38], and prediction of meat quality traits [39,40,41].

The prediction of meat-eating quality traits is highly challenging due to the hurdles associated with low accuracy of estimated breeding values, inconsistency in technical ease of measurement in live animals, non-repeatable reproducibility of carcass data, and high costs of rapid generation of data from large scale consumer sensory panels [19]. While the n-3 LC-PUFA profile of lamb and beef can be nutritionally enhanced using rumen-protected dietary supplements and pasture-based feeding [42,43,44], several research findings [2,4] emphasized the need for the more permanent and cumulative genetic selection route for meat sheep producers to guarantee the consistency of their lamb products in order to meet consumer preferences and adapt to the dynamics of purchasing decisions based on meat-eating quality. Consumers prefer meat with low FMP, moderate IMF, and fatty acid composition with proportionately more of the health-promoting n-3 LC-PUFA [45,46]. Since humans and other vertebrates lack Δ15 desaturase enzyme to synthesize n-3 LC-PUFA, they must obtain these from dietary intake sources in order to meet their daily requirement of 500 mg of n-3 LC-PUFA [4]. Therefore, lamb producers can tap into the omega-3 functional meat market niche through novel strategies for developing healthy meat products and reducing saturated fats [47] by matching their sheep breeding and production system to meet this health-conscious consumer preference [2,4].

### 4.1. SCD Gene Polymorphism

The *SCD* gene increases the desaturation of stearic acid to oleic acid and a functional variant in the *SCD* gene promoter affects fattening performance, carcass traits, meat quality, blood metabolites, and gene expression in ovine muscle [48,49]. Our results herein showing that TAW lambs were all heterozygous at three *SCD* SNP loci g.23881050T>C, g.23883280G>A and g.23885910C>A in the parental, first, and second composite generations (Table 2), presents a hereditary pattern and genetic divergence from the homozygous variants seen in the Poll Dorset, Texel, and Rambouillet controls that can be used as molecular markers for breed-specific identification. The significant correlations (Figure 7) and associations (Table 5 and Table 6) between detected *SCD* SNP loci, several fatty acids, and other meat-eating quality traits in TAW sheep is in consonance with other studies in Bashby × Argali [50], Rasa Aragonesa [49], Iranian fat- and thin-tailed [51], Poll Dorset × Border Leicester × Merino [52], Spanish, French, Egyptian, and Israeli sheep breeds [53] and Spanish goats [54]. In a comprehensive review of the genetics of n-3 LC-PUFA metabolism and meat-eating quality in TAW lambs [4], it was reported that although they were renowned for an outstanding low fat melting point (28–39 °C), high n-3 LC-PUFA EPA + DHA content (33–69 mg/100 g), marbling (3.4–8.2%), tenderness (20.0–38.5 N), and overall consumer liking (7.9–8.5), correlations between n-3 LC-PUFA profile, *SCD*, *FABP4*, *FASN*, and other lipogenic genes and meat quality traits presented major knowledge gaps. Therefore, the significant differences and associations observed at the *SCD g.23881050T>C* SNP locus in the present study where TAW lambs with the TT genotype had the highest DHA, IMF, and DPA compared to CC and CT genotypes (Table 6), have not only filled these knowledge gaps, but also equip lamb producers at the farmgate level to use this locus as a molecular marker for selection and breeding targeted at improving marbling and health-beneficial n-3 LC-PUFA. Since IMF in lamb has a moderately high heritability of 0.32–0.48 [55], has a direct relationship with tenderness, juiciness, and flavor [56] and surpasses the minimum acceptable consumer satisfaction threshold of 4% [57], the TAW lamb is well-positioned for a rapid genetic improvement for these meat-eating quality traits using the *SCD* gene g.23881050T>C SNP locus for identifying lambs at an early age.

### 4.2. FASN Gene Polymorphism

*FASN* catalyzes the synthesis of fatty acids such as palmitate from acetyl-CoA and malonyl-CoA, in the presence of NADPH, into long-chain saturated fatty acids, hence its involvement with fat deposition and fatty acid synthesis [58]. While novel genetic polymorphisms and gene expressions associated with carcass traits in Texel [59] and Rasa Aragonesa [60] sheep have been published, Sanz et al. [61] reported that only few studies focused on genetic variation in 5′ regulatory regions of genes involved in fat synthesis and metabolism pathways that could be good candidate genes. They went on to identify *FASN* gene polymorphisms and the potential use of these variants as markers associated with fat-related traits in Assaf, Roja Mallorquina, and Rasa Aragonesa sheep breeds [61]. In TAW sheep, our current study was the first to report significant genotype variations at the *FASN g.12323864A>G* SNP locus associated with FMP, ALA, MUFA, SFA, C18:2n-6, C18:1n-9, C18:0, and C16:0, in which the homozygous genotype GG had the highest values compared to the lowest values in AA genotype, with the exception of C18:2n-6 that was lowest in the heterozygous GA genotype (Figure 8, Table 5 and Table 6). This finding fills in a significant knowledge gap in sheep where very little has been reported on *FASN* gene, in stark contrast to many publications in cattle [62,63,64,65] and pigs [66,67,68,69]. Since fatty acid compositions determine the melting point and quality of fat and are closely related to the nutrition and meat-eating quality of lambs [70], our findings will assist TAW lamb producers to select the *FASN* genotypes best suited to their environments, market specifications, and processing needs in achieving efficiency in their management operations aimed at meeting consumer demand for healthy and nutritious lamb eating quality.

### 4.3. FABP Gene Polymorphism

The proteins of the *FABP4* family are small molecular-weight proteins that have a high binding affinity for long-chain fatty acids, participate in fatty-acid transportation from the plasma membrane to the sites of β-oxidation, triacylglycerol, and phospholipid synthesis, and variation in *FABP4* gene has been reported to affect fat deposition, growth, and meat production in sheep [71,72]. Several other research findings in sheep have demonstrated that dietary manipulation of omega-3 fatty acids can influence intramuscular fat deposition, growth, milk, wool, and meat quality [42,73,74,75,76,77,78,79,80,81,82,83,84,85,86,87,88,89,90], while only a handful of studies [52,91,92,93] have validated independent associations of carcass quality, shear force, intramuscular fat percentage, and omega-3 polyunsaturated fatty acid content with gene markers or the expression of genes encoding enzymes regulating fat metabolism in Australian lamb. Therefore, our current findings at the *FABP4* g.62829478A>- SNP locus showing consistently positive correlations between IMF and n-3 LC-PUFA of up to 0.66 with DHA, 0.47 with DPA, and 0.38 with EPA (Figure 9 and Table 5 and Table 6) provides a novel molecular marker for TAW sheep producers to select and breed lambs that are not only of high meat-eating quality, but also provide a healthy product for brain growth and development. This stems from the fact that IMF provides the needed marbling for taste, juiciness, and tenderness, while DHA being the major prevalent fatty acid in the brain membrane, is vital for the maintenance of healthy and functional brain development in infants and adults [94]. In pigs, Shang et al. [95] identified 3 FABP gene SNP and demonstrated that the genotype C-1375G was associated with fat deposition, while Gao et al. [96] reported that an association analysis of FABP SNP indicated that the polymorphism had a significant effect on marbling, in which pigs with the DD genotype had higher marbling than CD and CC genotypes, but the difference between CD and CC genotypes was not significant. They also reported that this FABP SNP had a highly significant effect on intramuscular fat content (*p* < 0.01). Our current study is the first report in TAW, which provides foundational data for the selection and breeding of lambs for marbling and healthy n-3 LC-PUFA using the identified SNP herein.

## 5. Conclusions

This study has provided novel insights into the shared genetic control of the fat melting point, intramuscular fat content, and health-beneficial omega-3 long-chain fatty acid composition traits that are helpful in designing breeding strategies to genetically improve meat-eating quality traits in TAW lambs while they are still alive. The early decision making utilizing this innovative and minimally invasive *longissimus dorsi thoracis et lumborum* muscle biopsy sampling technique for directly quantifying the genetic worth of live lambs overcomes the problem of waiting to collect meat quality data after slaughter when selection decisions about the live animal are already too late. As the present data are laboratory-tested, personalized, and customized to actual individual lamb performance and not based on estimated breeding values, precision problems due to low accuracy are minimized. The identified SNP of these lipid metabolism genes can also be used for breed-specific identification and marker-assisted selection of Tattykeel Australian White (TAW) sheep exclusive to MARGRA lamb brand for high-end meat-eating quality. Next-generation sequencing of the *FABP4*, *FASN,* and *SCD* genes also provides foundational data underpinning their roles in fatty acid metabolism unique to the TAW breed.

## Figures and Tables

**Table 1:** Primer sequences for *FABP4*, *FASN,* and *SCD* polymerase chain reaction assays ^#^.

Gene		Sequence	Length (bp)	T_a_ (°C)	Fragment Length (bp)
*FASN 1*	Forward	CCTACTTTCCCATGCTCAGAGAA	23	68	7890
	Reverse	CTACGTTGCTGAGGAAGAACTCTA	24	68	
*FASN 2*	Forward	ACCGTCTCTCCTTCTTCTTTGAC	23	68	8798
	Reverse	GAAGTTGAGGGAGGCGTAATAGAT	24	68	
*FASN 3*	Forward	CTAGAGTTCTTCCTCAGCAACGTA	24	68	9288
	Reverse	GCCAGGGAGCTGTGAATAATACTA	24	68	
*FABP4*	Forward	TTGTTGAATGGCTGGGCTTATAAC	24	60	4107
	Reverse	TAAGAAAATACTTCCTGGGGCACA	24	60	
*SCD*	Forward	CAAACTTAGGTCTGCAACTTTCGT	24	65	11,545
	Reverse	TTTCCCACTTCAACTCACCCTATT	24	65	

^#^*FASN*, Fatty Acid Synthase; *FABP4*, Fatty Acid Binding Protein 4; *SCD*, Stearoyl-CoA Desaturase; T_a_, annealing temperature.

**Table 2:** *SCD* gene SNP (major allele frequency) in TAW ^1^, Poll Dorset (+control), and Rambouillet (−control) lambs.

Lamb breed, generation, type of control and genotypes (major allele frequencies in brackets) Parental composites 1st (F1) and 2nd (F2) composites Positive (+) and negative (−) controls						
**SNP Locus**	**TAW Parents** **(*n* = 147)**	**TAW F1** **(*n* = 75)**	**TAW F2** **(*n* = 75)**	**Poll Dorset** **(+*n* = 2)**	**Texel** **(+*n* = 2)**	**Rambouillet** **(−*n* = 2)**
g.23880613A>G	GG (0.82)	GG (0.93)	GG (0.73)	GG	GG	AA
g.23881050T>C	CT (0.58)	CT (0.54)	CT (0.90)	CC	CC	TT
g.23883280G>A	AG (0.53)	AG (0.71)	AG (0.60)	AA	AA	GG
g.23885910C>A	AC (0.57)	AC (0.71)	AC (0.53)	CC	CC	CC
g.23887165A>G	GA (0.69)	GG (0.82)	GG (0.70)	GG	GG	AA
g.23888763C>T	TC (0.58)	TC (0.54)	CC (0.93)	CC	CC	CC
g.23889346T>G	GT (0.68)	GG (0.82)	GG (0.70)	GG	GG	TT
g.23890209T>C	CT (0.67)	CC (0.82)	CC (0.70)	CC	CC	TT

^1^ TAW, Tattykeel Australian White.

**Table 3:** *FASN* gene SNP (major allele frequency) in TAW ^1^, Poll Dorset (+control) and Rambouillet (−control) lambs.

Lamb breed, generation, type of control, and genotypes (major allele frequencies in brackets) Parental composite 1st (F_1_) and 2nd (F_2_) composites, Positive (+) and negative (−) controls						
**SNP Locus**	**TAW Parents** **(*n* = 147)**	**TAW F_1_** **(*n* = 75)**	**TAW F_2_** **(*n* = 75)**	**Poll Dorset** **(+*n* = 2)**	**Texel** **(+*n* = 2)**	**Rambouillet** **(−*n* = 2)**
g.12316077T>G	GG (0.89)	GG (0.86)	GG (0.95)	GG	GG	TT
g.12318491A>G	GG (0.89)	GG (0.86)	GG (0.95)	GG	GG	AA
g.12320583T>C	CC (0.89)	CC (0.86)	CC (0.97)	CC	CC	TT
g.12321671T>C	CC (0.89)	CC (0.86)	CC (0.97)	CC	CC	TT
g.12323864A>G	GA (0.70)	GA (0.69)	GA (0.70)	GG	GG	AA
g.12324288G>A	AG (0.69)	AG (0.68)	AG (0.69)	AA	AA	GG
g.12326992T>C	CC (0.88)	CC (0.79)	CC (0.90)	CC	CC	TT
g.12327084->CT	CT (0.50)	CT (0.50)	CT (0.50)	CT	CT	TT
g.12328120T>C	CC (0.89)	CC (0.86)	CC (0.97)	CC	CC	TT

^1^ TAW, Tattykeel Australian White.

**Table 4:** *FABP4* gene SNP (major allele frequency) in TAW ^1^, Poll Dorset (+control) and Rambouillet (−control) lambs.

Lamb breed, generation, type of control, and genotypes (major allele frequencies in brackets) Parental composites 1st (F_1_) and 2nd (F_2_) composites Positive (+) and negative (−) controls						
**SNP Locus**	**TAW Parents** **(*n* = 147)**	**TAW F_1_** **(*n* = 75)**	**TAW F_2_** **(*n* = 75)**	**Poll Dorset** **(+*n* = 2)**	**Texel** **(+*n* = 2)**	**Rambouillet** **(−*n* = 2)**
g.62826961T>C	CT (0.61)	TT (0.64)	CT (0.60)	TT	TT	TT
g.62826965C>G	GC (0.61)	GC (0.57)	GC (0.60)	GG	GG	CC
g.62829478A>T	AT (0.55)	AT (0.61)	AT (0.53)	AA	AA	AA

^1^ TAW, Tattykeel Australian White.

**Table 5:** Associations between SNP mutations and FMP, IMF, and fatty acids in TAW lambs ^#^.

SNP effect (*p*-values) *SCD FABP4 FASN*						
**Variable**	**Mean**	**SD**	**CV (%)**	**g.23881050T>C**	**g.62829478A>T**	**g.12323864A>G**
FMP (°C)	33.65	2.74	8.14	0.2700	0.6115	0.0544 *
IMF (%)	4.43	1.31	29.58	0.0089 **	0.0539 *	0.1915
*Fatty acids (mg/100 g)*						
ALA (C18:3n-3)	163.03	192.27	117.94	0.7755	0.1419	0.0033 **
EPA (C20:5n-3)	25.20	11.62	46.10	0.7683	0.1023	0.9810
DHA (C22:6n-3)	8.43	4.16	49.27	0.0111 *	0.2145	0.9480
DPA (C22:5n-3)	23.85	13.70	57.44	0.0532 *	0.3894	0.0927
EPA + DHA	33.64	14.75	43.84	0.2036	0.4794	0.9915
EPA + DHA + DPA	57.49	26.97	46.92	0.0728	0.8958	0.2004
MUFA	3694.70	4099.08	110.94	0.6824	0.3949	0.0025 **
SFA	4392.18	5238.81	119.28	0.4000	0.5472	0.0029 **
C18:2n-6	253.68	247.70	97.64	0.6781	0.0647	0.0138 *
C14:0	287.92	437.58	151.98	0.0632	0.7354	0.1190
C16:0	2076.17	2419.46	116.53	0.5414	0.3751	0.0039 **
C18:0	1683.83	2065.71	122.68	0.3891	0.9125	0.0012 **
C18:1n-9	2901.10	3212.65	110.74	0.8555	0.3696	0.0023 **

^#^* *p* < 0.05, ** *p* < 0.01; SFA, Saturated fatty acids; MUFA, Monounsaturated fatty acids; SD, Standard Deviation; CV, Coefficient of variation.

**Table 6:** Tukey-adjusted multiple comparisons between SNP mutations and FMP, IMF, and fatty acids in TAW lambs ^#^.

Multiple Genotype Comparisons						
**SNP Locus**	**Variable**	**Mean ± SE**	**Genotypes**		**Difference ± SE**	***p*-Value**
***SCD g.23881050T>C***	*DHA (C22:6n-3)* (mg/100 g)					
	CC	7.00 ± 2.11	CC vs.	CT	−0.639 ± 0.834	0.7247
	CT	7.64 ± 2.09	CC vs.	TT	−3.998 ± 1.334	0.0105 *
	TT	11.00 ± 2.34	CT vs.	TT	−3.359 ± 1.235	0.0223 *
	*IMF* (%)					
	CC	3.98 ± 0.312	CC vs.	CT	−0.407 ± 0.323	0.4224
	CT	4.39 ± 0.287	CC vs.	TT	−1.446 ± 0.532	0.0222 *
	TT	5.43 ± 0.516	CT vs.	TT	−1.038 ± 0.502	0.1041
	*DPA (C22:5n-3)* (mg/100 g)					
	CC	17.9 ± 6.81	CC vs.	CT	−1.56 ± 2.65	0.8270
	CT	19.4 ± 6.74	CC vs.	TT	−9.19 ± 4.25	0.0850
	TT	27.1 ± 3.26	CT vs.	TT	−7.63 ± 3.93	0.0356 *
***FASN g.12323864A>G***	*FMP* (°C)					
	GG	34.2 ± 0.4	GG vs.	GA	0.81 ± 0.64	0.4201
	GA	33.4 ± 0.3	GG vs.	AA	2.98 ± 1.61	0.0536 *
	AA	31.5 ± 1.5	GA vs.	AA	2.16 ± 1.60	0.3685
	*ALA (C18:3n-3)* (mg/100 g)					
	GG	188.7 ± 67.6	GG vs.	GA	114.7 ± 39.9	0.0149 *
	GA	74.0 ± 66.7	GG vs.	AA	147.2 ± 100.1	0.3115
	AA	41.5 ± 113.7	GA vs.	AA	32.6 ± 99.8	0.9430
	*MUFA* (mg/100 g)					
	GG	4524 ± 1384	GG vs.	GA	2617 ± 867	0.0099 **
	GA	1907 ± 1361	GG vs.	AA	3089 ± 2175	0.3363
	AA	1436 ± 2415	GA vs.	AA	472 ± 2168	0.9742
	*SFA* (mg/100 g)					
	GG	5479 ± 1715	GG vs.	GA	3270 ± 1121	0.0132 *
	GA	2208 ± 1684	GG vs.	AA	4162 ± 2812	0.3068
	AA	1317 ± 3086	GA vs.	AA	892 ± 2803	0.9458
	*C18:2n-6* (mg/100 g)					
	GG	281 ± 84.8	GG vs.	GA	142.5 ± 52.2	0.0216 *
	GA	139 ± 83.4	GG vs.	AA	105.8 ± 130.8	0.6988
	AA	175 ± 146.4	GA vs.	AA	−36.7 ± 130.4	0.9573
	*C16:0* (mg/100 g)					
	GG	2539 ± 800	GG vs.	GA	1475 ± 518	0.0158 *
	GA	1063 ± 786	GG vs.	AA	1826 ± 1298	0.3433
	AA	713 ± 1429	GA vs.	AA	350 ± 1294	0.9604
	*C18:0* (mg/100 g)					
	GG	2227 ± 658	GG vs.	GA	1419 ± 441	0.0056 **
	GA	809 ± 646	GG vs.	AA	1711 ± 1106	0.2756
	AA	516 ± 1205	GA vs.	AA	292 ± 1102	0.9620
	*C18:1n-9* (mg/100 g)					
	GG	3589 ± 1078	GG vs.	GA	2103 ± 679	0.0080 **
	GA	1486 ± 1060	GG vs.	AA	2353 ± 1704	0.3566
	AA	1236 ± 1892	GA vs.	AA	250 ± 1698	0.9882
***FABP4* g.62829478A>T**	*IMF* (%)					
	A	4.57 ± 0.39	A vs.	AA	0.07 ± 0.344	0.0556
	AA	3.92 ± 0.39				

^#^* *p* < 0.05, ** *p* < 0.01; SFA, Saturated fatty acids; MUFA, Monounsaturated fatty acids; SD, Standard Deviation; CV, Coefficient of variation.

## Data Availability

Data available on request.
